# A cloud-edge adaptive lightweight network with dynamic inference enables real-time ultrasound image segmentation

**DOI:** 10.1038/s41598-026-59234-y

**Published:** 2026-06-29

**Authors:** Chaoyu Li, Jiexia Tan

**Affiliations:** 1 Faculty of Electronic and Information Engineering, Xi’an Jiaotong University, Xi’an, 710049 China; 2 School of Electronic Information Engineering, Wuzhou Vocational College, Wuzhou, 543000 China

**Keywords:** Ultrasound image segmentation, Lightweight network, Cloud-edge collaboration, Dynamic inference, Real-time segmentation, Computational biology and bioinformatics, Engineering, Health care, Mathematics and computing

## Abstract

Ultrasound image segmentation plays an important role in clinical diagnosis, but existing deep learning methods struggle to balance accuracy and efficiency, limiting practical deployment in resource-constrained medical scenarios. This study proposes a Cloud-Edge Adaptive Lightweight Network (CEA-Net) with dynamic inference, implementing parameter compression through a lightweight encoder constructed with depthwise separable convolutions and inverted residual structures, introducing serial channel-spatial attention mechanisms to enhance lesion feature representation, and designing intelligent routing algorithms based on sample complexity perception to dynamically select edge fast inference or cloud enhanced processing paths according to ultrasound image complexity. Experimental validation on the BUSI breast ultrasound dataset and TN3K thyroid nodule dataset demonstrates that CEA-Net significantly reduces model parameters and inference latency while maintaining high segmentation accuracy, achieves near real-time inference capability on edge devices, and reaches a favourable balance between accuracy and speed through intelligent task allocation in cloud-edge collaborative mode. This study provides a feasible technical solution for practical deployment of ultrasound image segmentation technology in resource-constrained scenarios including primary healthcare institutions, telemedicine, and mobile diagnosis, advancing the practical application of medical artificial intelligence technology from cloud to edge.

## Introduction

Ultrasound image technology has become an important part of clinical practice as a medical imaging modality due to its non-invasive nature, real-time imaging ability, and cost-effectiveness. It has played an irreplaceable role in the diagnosis of various diseases, such as breast cancer and thyroid nodules. In recent years, the development of deep learning technology has provided new avenues for the segmentation of medical ultrasound images. Review study have systematically reviewed the frontier development and challenges of deep learning-based medical image/video segmentation technology^1^, and the segmentation technology of B-mode ultrasound images has achieved significant breakthroughs based on convolutional neural networks and Transformer networks^2^. Artificial intelligence technology is revolutionizing the application of medical image technology in an unprecedented way^3^, but a notable gap still exists in the trade-off between the high computational complexity of traditional deep learning models and the limited resources of medical applications. Although the problem of insufficient data in medical image segmentation can be alleviated by semi-supervised learning frameworks such as shadow-aware networks and boundary refinement techniques^4^, the efficiency of these networks is still insufficient to meet the requirements of real-time dynamic scanning. In recent years, various segmentation algorithms of thyroid ultrasound images have improved the accuracy of thyroid nodules by optimizing U-Net structures^5^, and multi-scale feature fusion and hierarchical attention mechanisms have shown potential in the three-dimensional visualization of thyroid ultrasound images^6^, but these networks are commonly confronted with the problem of excessive parameters and are difficult to be deployed in edge devices.

Research on attention mechanisms in medical image segmentation has progressed from channel attention to various methods of feature enhancement in multiple dimensions, and relevant reviews have comprehensively discussed the application status and development trends of attention mechanisms in medical image segmentation^7^. Recent studies have also proved that efficient attention mechanisms play a vital role in improving image segmentation performance^8^, and innovative 3D medical image segmentation networks have achieved a balance between efficiency and accuracy by using decoupled encoder-decoder structures in image segmentation networks^9^. The emergence of large-scale vision foundation models has created new technical paths for universal image segmentation in the field of medicine^10^, although it is affected by the large parameter scale, which limits its application in resource-constrained devices. The introduction of hybrid attention mechanisms has enhanced feature representation capabilities by combining information in both spatial and channel dimensions^11^, and large kernel attention has shown unique advantages in 3D medical image processing^12^. The introduction of multi-focal channel attention has greatly enhanced the perception of critical areas in image segmentation^13^, and deep learning has been optimized in breast tumor lesion segmentation^14^. Although these studies have achieved better performance in image segmentation, they have pursued performance maximization while ignoring efficiency, making it difficult to meet the requirements of real-time diagnosis.

The development of lightweight network architectures offers opportunities to address the problem of computational efficiency. MobileNets have greatly reduced the complexity of models by using depthwise separable convolution^15^, opening up opportunities for mobile vision tasks. EfficientFormer uses the power of the Transformer network in a lightweight way to achieve inference speed close to that of MobileNet^16^. Structural reparameterization technology has been used in MobileOne to achieve millisecond-level inference speed^17^, while tremendous potential for the compression of vision Transformers’ inference in mobile devices has been found^18^. The hybrid network of convolutional networks and Transformers has shown unique advantages in mobile applications^19^, while FPGA acceleration technology provides hardware-level support for real-time inference in resource-constrained environments^20^. Medical image segmentation networks optimized for edge devices have been proposed to dramatically reduce parameters while ensuring segmentation accuracy by using lightweight U-Net structures^21^. However, existing lightweight networks have compromised segmentation accuracy in favor of model compression and lack the ability to adapt to the dynamic change in the complexity of ultrasound images.

The development of the edge computing paradigm has opened up new opportunities for medical image analysis. The integration of deep learning and edge computing has accelerated the development of distributed intelligent processing architectures^22^. Efficient training methods in Internet of Medical Things environments facilitate the participation of edge devices in optimization training processes^23^. Blockchain-assisted resource allocation mechanisms offer reliable task scheduling solutions for edge computing environments^24^. Collaborative inference methods based on the decoupling of deep neural networks facilitate multi-agent cooperation through intermediate feature compression^25^. Cloud-edge collaborative environments have verified the practicability of distributed inference methods for COVID-19 diagnosis scenarios^26^. Medical image analysis methods based on the interaction between multiple features have improved the capabilities of image processing through cloud computing^27^. Collaborative inference methods for mobile edge computing environments optimize overall performance through task offloading^28^. Cloud-magnetic resonance imaging environments in the 6G era demonstrate the vast potential for cloud-edge integration^29^. Cloud-edge collaborative environments for ICU scenarios realize multi-device semantic interoperability^30^. However, existing research focuses mainly on static task allocation, without sufficient consideration of the impact of dynamic changes in the complexity of medical images on collaborative inference methods.

With the introduction of dynamic inference mechanisms, new insights have been provided for the realization of efficient inference through the adaptive selection of computational paths. The concept of early exiting strategies enables simple samples to finish the inference process at a shallow layer of the network, thus preventing wastage of computation^31^. The concept of dynamic routing learning enables the adaptive selection of processing paths according to the features of the input samples^32^. The study of dynamic neural networks has provided insights into the advantages and challenges of adaptive inference, which can reduce the costs of computation^33^. The spatial sparsification technology has provided a boost to the efficiency of vision models by eliminating wastage of features^34^. The post-deployment adaptation of neural architecture can dynamically adjust the structure of the model according to the heterogeneity of the environment^35^. The existing dynamic inference research has only considered single-device optimization and has not utilized the advantages of the complementarity of resources in cloud and edge environments to address the large variation of complexity in ultrasound images.

Comprehensive analysis of existing research reveals three fundamental insufficiencies. Deep learning segmentation methods and lightweight network design lack organic integration, resulting in sacrificing computational efficiency when pursuing segmentation accuracy or losing segmentation performance when compressing model size. Edge computing research mostly focuses on static deployment optimization and fails to design adaptive inference strategies targeting the dynamic variation of ultrasound image quality and lesion complexity. Dynamic inference mechanisms are limited to computational resource scheduling on single devices and fail to fully exploit the potential of cloud-edge collaborative architectures in heterogeneous resource management and intelligent task allocation.

The preceding limitations acquire particular relevance in ultrasound imaging, whose formation process distinguishes it from the higher signal-to-noise modalities such as computed tomography and magnetic resonance imaging on which many segmentation networks are developed. Coherent wave interference introduces speckle, acoustic attenuation produces weak contrast between lesions and surrounding soft tissue, and acoustic shadowing leaves boundaries diffuse or partially occluded, so that the discriminative cues are subtle and prone to loss under aggressive parameter compression, which makes the accuracy-efficiency conflict identified above harder to resolve in ultrasound than in those higher-contrast modalities and favours lightweighting that preserves low-contrast boundary detail rather than compression applied indiscriminately. The quality variation noted above arises from the acquisition process itself, since operator dependence, probe orientation, and patient anatomy cause image quality to range from clear regular lesions to noticeably degraded acquisitions, while point-of-care deployment places the processing on hardware whose computational budget is well below that of a workstation, so that a single fixed computational path cannot serve easy and difficult acquisitions with comparable efficiency. The dependence of segmentation difficulty on these modality-specific properties is the reason the present work is developed and evaluated on dedicated ultrasound benchmarks rather than on generic natural-image datasets, since a viable solution should be calibrated to the noise, contrast, and quality-variation profile of ultrasound rather than to general object boundaries.

This study proposes a Cloud-Edge Adaptive Lightweight Network (CEA-Net) for real-time ultrasound image segmentation, providing an effective technical solution for deployment in primary healthcare institutions and mobile diagnostic scenarios. The principal contributions of this study are summarized as follows.

- A lightweight encoder built from depthwise separable convolutions and inverted residual blocks is designed for ultrasound feature extraction, reducing the parameter count to a level suitable for edge deployment while preserving the fine-grained discrimination required under speckle and low lesion contrast.
- A serial channel-spatial attention fusion decoder employing a shared multilayer perceptron and a low-rank bottleneck is developed to strengthen boundary representation for low-contrast lesions without incurring the parameter overhead typical of multi-dimensional attention modules.
- A sample complexity perception module coupled with an intelligent routing algorithm is proposed to quantify per-image difficulty and to allocate simple cases to an edge fast path and complex cases to a cloud enhanced path, forming a cloud-edge adaptive dynamic inference mechanism that exploits heterogeneous resources rather than scheduling computation on a single device.
- Experiments on the BUSI and TN3K datasets validate the effectiveness of the proposed network and its balance among segmentation accuracy, inference latency, and model complexity under both cloud-only and cloud-edge collaborative deployment.

## Methodology

### CEA-net overall architecture

**Fig. 1:**
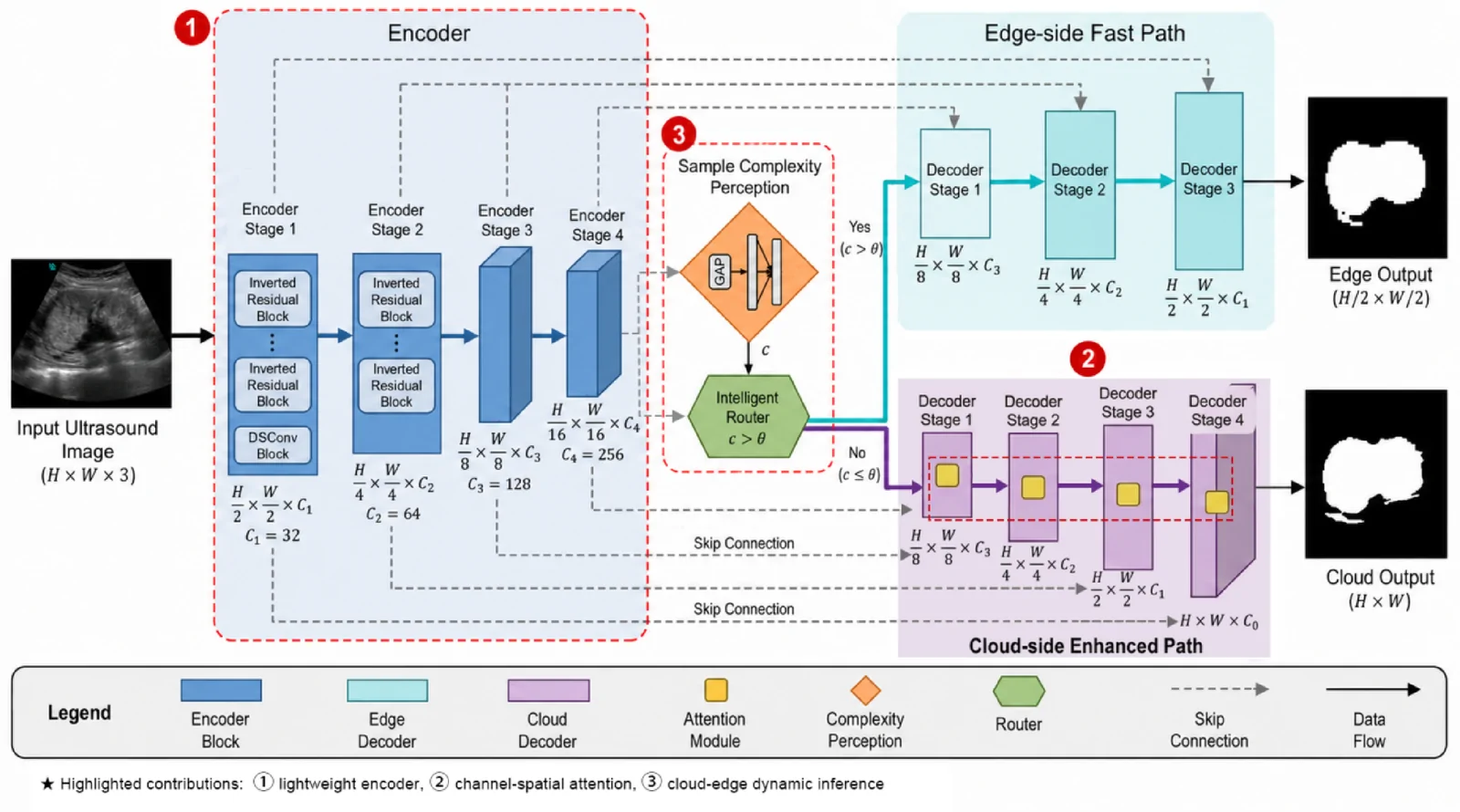
Overall architecture of the proposed CEA-Net.

The CEA-Net utilizes an encoder-decoder architecture to facilitate real-time ultrasound image segmentation within the cloud-edge collaboration environment. The core philosophy of the entire system is to adaptively allocate the computing resources based on the complexity characteristics of the ultrasound images, where simple cases are segmented by the edge-side lightweight path, while complex cases are segmented by the cloud-side enhanced path. The architecture of the proposed CEA-Net includes five major components, which are a lightweight encoder for multi-scale feature extraction, a sample complexity perception module for evaluating the complexity, an intelligent routing decision unit for adaptive routing, a dual-branch inference unit for the edge-side rapid processing path and the cloud-side enhanced processing path, and an attention-based fusion decoder for the integration of the multi-scale features to generate the final segmentation results. The input ultrasound images are first processed by the lightweight encoder for the extraction of the multi-scale features, which are then sent to the complexity evaluation network to generate the confidence score. If the confidence score is higher than a predetermined threshold, the edge-side rapid processing path is invoked, otherwise, the processing task will be sent to the cloud for the enhanced processing path. In order to clearly describe the entire workflow of the proposed CEA-Net, the entire computation process for the ultrasound image segmentation, including the critical decision points for the cloud-edge collaboration, is illustrated in Fig. 1.

As can be seen from the architecture diagram in Fig. 1, the encoder’s four downsampling stages compress the input from $H \times W \times 3$ to $\frac{H}{16} \times \frac{W}{16} \times C_{4}$ with the features of each stage transmitted to the decoder via skip connections. The dynamic routing decision module makes the selection of the inference path based on the statistical characteristics of the features. The outputs of the edge-side path are obtained at the third decoding stage for the purpose of quick inference, while the outputs of the cloud-side path pass through the four stages with attention enhancement added to each stage for higher boundary precision.

The depthwise separable convolutions, inverted residual blocks, and channel-spatial attention adopted in CEA-Net are established building blocks, so the distinctive contribution lies not in these operators but in the way they are organized into a complexity-aware cloud-edge system. Rather than partitioning the network statically, a single lightweight encoder serves both inference paths, and the intelligent router allocates each acquisition to the edge or the cloud according to its estimated difficulty, so that computation is matched to per-image complexity. The two paths share this encoder and differ only in decoder depth and in whether attention refinement is applied, with the routing and offloading mechanism detailed in “Cloud-edge adaptive dynamic inference mechanism” section, so that the collaboration adds little beyond the baseline segmentation network. This difficulty-aware allocation across heterogeneous cloud and edge resources, rather than any individual operator, constitutes the distinctive advantage of the proposed method.

### Lightweight encoder design

The lightweight encoder possesses the capability of feature extraction while greatly reducing the number of parameters through the use of depthwise separable convolution and inverted residual structure. In traditional standard convolution, both spatial feature extraction and channel feature fusion are combined in one operation, and the computational complexity increases linearly with the multiplication of input and output channels. In depthwise separable convolution, the two operations of spatial feature extraction and channel feature fusion are separated, where depthwise convolution is used for spatial feature extraction of each channel of the input, and then pointwise convolution is used for linear combination of channels. The research on crowd density estimation using edge intelligence has proved the validity of lightweight convolutional neural networks for use in resource-scarce devices^36^, but the characteristics of low contrast between lesions and background tissue in ultrasound images require that the networks have stronger fine-grained feature discrimination capability, which requires structural optimization of the networks according to the characteristics of speckle noise and boundary blurring in ultrasound images.

**Fig. 2:**
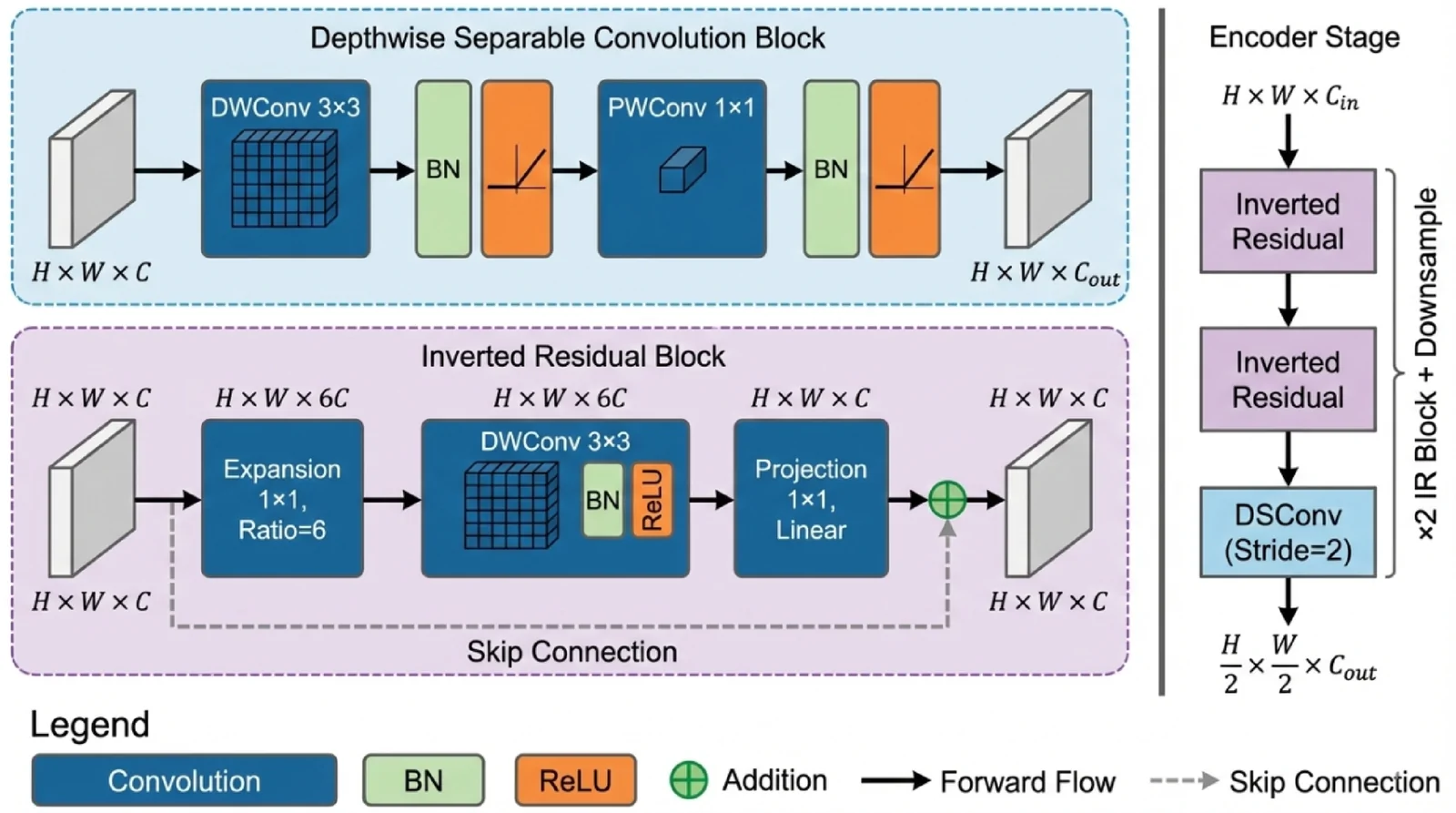
Detailed structure of lightweight encoder with depthwise separable convolution and inverted residual blocks.

The encoder has four stages of progressive downsampling, with each stage consisting of two inverted residual blocks and one depthwise separable convolution with stride 2 for downsampling. In the inverted residual block, there is an "expansion-convolution-projection" three-layer structure, with the channel expansion ratio being set to 6, so shortcut connections between low-dimensional input and output, as well as nonlinear transformation, are established in the high-dimensional space, avoiding the destructive clipping effect of the ReLU activation function. The output feature map dimensions at each stage are $H/2 \times W/2 \times C_{1}$, $H/4 \times W/4 \times C_{2}$, $H/8 \times W/8 \times C_{3}$, and $H/16 \times W/16 \times C_{4}$ respectively, with channels increasing according to [32, 64, 128, 256]. To elaborate on the internal computational flow and parameter compression strategy of the lightweight encoder, Fig. 2 demonstrates the detailed structural decomposition of depthwise separable convolution modules and inverted residual blocks, with comparative annotations of parameter counts and computational complexity for each operation.

The depthwise separable convolution module shown in Fig. 2 contains a sequence of *3 × 3* depthwise convolution, batch normalization, ReLU activation, and *1 × 1* pointwise convolution, with parameter count reduced from $C_{in} \times C_{out} \times k^{2}$ in standard convolution to $C_{in} \times k^{2} + C_{in} \times C_{out}$. The inverted residual block expands channels from *C* to 6C through *1 × 1* expansion convolution, extracts spatial features via *3 × 3* depthwise convolution, and compresses back to *C* dimensions through *1 × 1* projection convolution. Together, these two operations give the encoder an edge-deployable parameter footprint, which forms the lightweight-encoder contribution of this study.

### Cloud-edge adaptive dynamic inference mechanism

The cloud edge adaptive dynamic inference mechanism is the core technology that differentiates CEA-Net from traditional deep neural networks in terms of intelligent management of computational resources through perception of sample complexity. In terms of efficient management of inferences in mobile and embedded systems, previous studies have proposed the framework for dynamic deep neural networks^37^. However, most studies have focused mainly on path skipping strategies in single-device heterogeneous systems without considering collaborative optimization in cloud edge heterogeneous systems. In terms of adaptive dynamic inferences with multi-route neural networks, path selection strategies with constraints have also been explored in previous studies^38^. However, most studies have focused on cloud edge heterogeneous systems as two mutually exclusive systems without considering collaborative optimization in heterogeneous systems. This study aims to propose a novel framework for collaborative inferences in heterogeneous systems, in which complex samples can complete preliminary inferences in edge devices before sending intermediate features to the cloud for further processing with superior accuracy-latency tradeoffs.

The sample complexity perception module receives the encoder’s final layer feature map $F_{4} \in {\mathbb{R}}^{{H/16 \times W/16 \times C_{4} }},$ extracting spatial dimension statistical information through global average pooling. The complexity scoring network adopts a lightweight two-layer fully connected structure, with the first layer compressing feature dimensions from $C_{4}$ to $C_{4} /4$ and applying ReLU activation, while the second layer maps to a single scalar output with Sigmoid activation normalizing the output to the *(0,1)* interval representing confidence score, calculated as

1 $$c = \sigma \left( {W_{2} \cdot \max (0,W_{1} \cdot {\mathrm{GAP}}(F_{4} ) + b_{1} ) + b_{2} } \right)$$

**Fig. 3:**
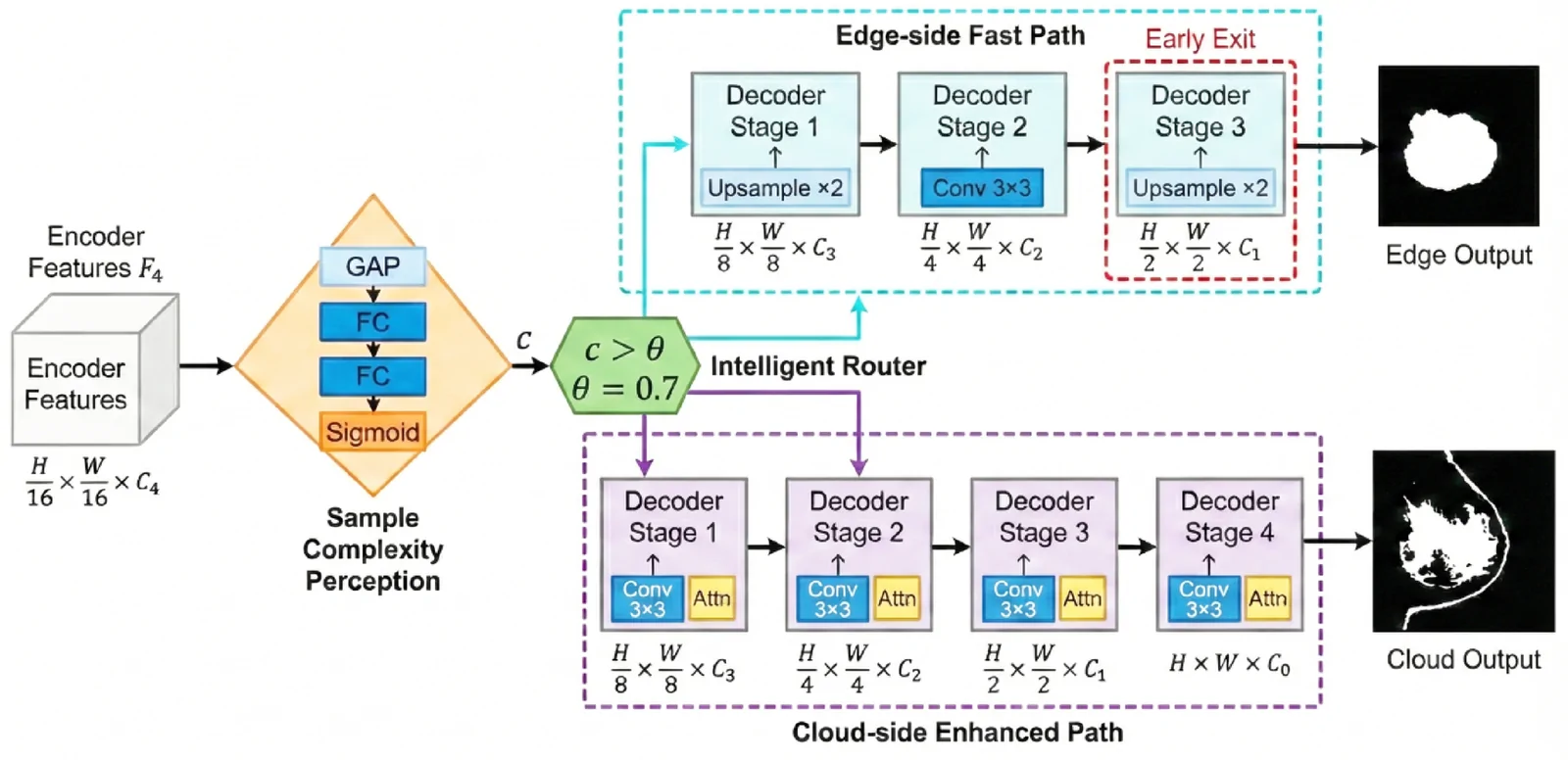
Cloud-edge adaptive dynamic inference mechanism with sample complexity perception.

The confidence score *c* reflects the probability that the current sample obtains satisfactory segmentation results at the edge. The threshold *θ* is determined by maximizing ${\mathrm{Dice}} - \lambda \times {\mathrm{Latency}}$ on the validation set, with experimental settings of *θ = 0.7* and *λ = 0.01*. When *c > θ*, samples are classified as simple cases directly entering the edge-side fast inference path; when $c \le \theta$, task offloading mechanisms are triggered to transmit encoder output features to the cloud for enhanced processing. To intuitively demonstrate the complete inference decision flow of cloud-edge collaboration and structural differences between dual paths, Fig. 3 presents the detailed computational topology from complexity evaluation and intelligent routing to edge-side and cloud-side branches.

The mechanism shown in Fig. 3 demonstrates that encoder features are processed through the complexity perception module containing sequential GAP, FC layers, and Sigmoid activation to generate confidence score *c*. The intelligent router compares *c* with threshold *θ = 0.7* to determine the inference path. The edge-side fast inference path contains only three decoding stages with early exit at *H/2 × W/2* resolution, while the cloud-side enhanced path contains complete four decoding stages with attention modules embedded at each stage to achieve higher boundary precision. The dual outputs illustrate the trade-off between computational efficiency and segmentation accuracy in the cloud-edge collaborative framework.

### Attention fusion and decoder module

The function of the attention fusion decoder is to combine multi-scale feature information learned by the encoder and gradually recover spatial resolution while improving expression of feature information in key areas through channel and spatial dual-dimension attention mechanisms. The study on medical image segmentation using multi-scale cross-axis attention has provided effective methods for cross-dimensional feature interaction^39^, which can achieve efficient modeling of 3D medical image information by calculating attention weights in different spatial dimensions, although it is mainly used in image segmentation in high signal-to-noise ratio imaging modalities such as CT and MRI scans, without specific anti-interference mechanisms for speckle noise and artifacts in ultrasound imaging. The study on 3D medical image segmentation using gated attention blocks and dual-scale cross-attention mechanism further proved the superiority of multi-dimensional attention fusion in image feature expression^40^, in which the gating mechanism can learn adaptive weights to control the proportion of feature information at different scales, which is helpful in designing the attention module in this study. However, it can be found that all these methods have a common drawback of too many parameters. This study has avoided the drawback of excessive parameters while retaining the advantages of channel-spatial joint modeling by using shared multilayer perceptron and low-rank decomposition strategies.

**Fig. 4:**
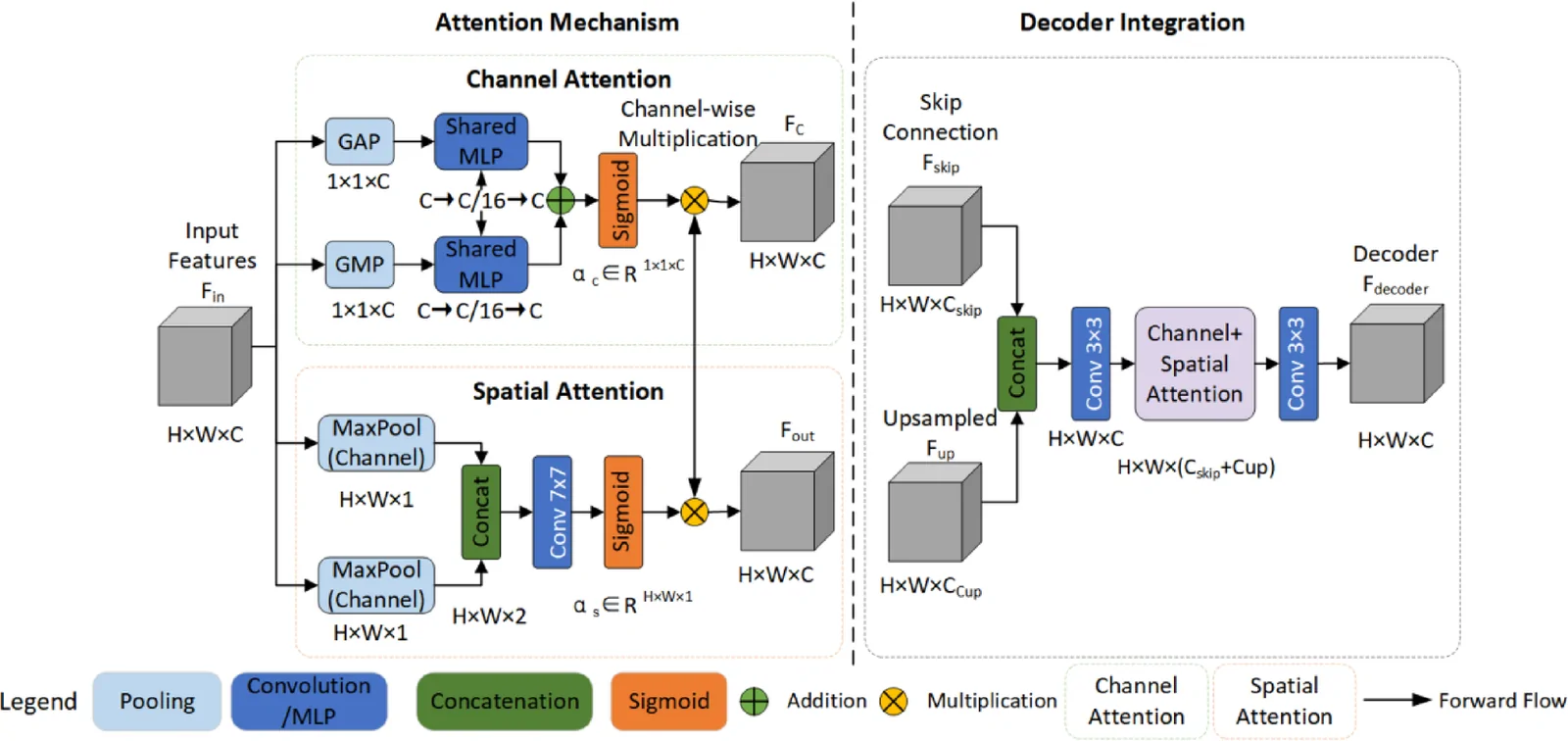
Attention fusion module integrating channel and spatial attention mechanisms.

The lightweight channel attention module uses global average pooling and global max pooling to obtain the mean and peak statistical information of the feature maps. The two pooling operations share the same multilayer perceptron network to avoid parameter redundancy. In the multilayer perceptron network, the number of channels in the intermediate layer is set to 1/16 of the input channels in a bottleneck structure. The calculation of channel attention weight is based not only on the average intensity of feature responses but also on the maximum responses. The spatial attention module performs max pooling and average pooling along the channel dimension followed by concatenation along the channel axis, modeling spatial dependencies within a larger receptive field through *7 × 7* convolution kernels, with Sigmoid activation generating spatial weight maps that highlight lesion regions while suppressing background interference. Serial application is used by channel and spatial attention. Channel attention filters the semantically relevant feature channels first, and then spatial attention focuses the regions of interest. The sequential application facilitates the network to enhance features from global semantics to local details. To describe the computational process of the attention modules and their integration with the decoder, Fig. 4 depicts the serial application of channel and spatial attention with multi-scale feature fusion methods.

The attention fusion module shown in Fig. 4 demonstrates the serial integration of channel and spatial attention mechanisms. The left panel illustrates that channel attention employs parallel GAP and GMP operations followed by a shared MLP with 1/16 compression ratio to generate channel weights $\alpha_{c}$, while spatial attention applies channel-wise max and average pooling followed by *7 × 7* convolution to produce spatial weights $\alpha_{s}$. The right panel shows the decoder integration, which involves the combination of the skip connection features from the encoder layers and the upsampling features from the preceding decoder stage, convolution, and the serial channel-spatial attention module for the final generation of decoder outputs. This parameter-efficient attention design constitutes the channel-spatial attention contribution of this study.

### Loss function design

The design of the loss function takes into account the three optimization objectives from a holistic perspective: region overlap, classification accuracy at the pixel level, and boundary refinement. The dice loss optimizes the parameters of the network by maximizing the ratio of the intersection and union between the predicted segmentation and the ground truth labels, and it has inherent robustness against class imbalance. The binary cross-entropy loss optimizes the classification accuracy at the pixel level by minimizing the divergence between the predicted and ground truth distributions. Because the lesion commonly occupies only a small fraction of an ultrasound image, a pixel-level binary cross-entropy term applied on its own would be dominated by the abundant background pixels, and this foreground–background imbalance is counteracted by the dice loss noted above, which is assigned the largest weight in the total objective in Eq. (3), so that the optimization remains sensitive to the under-represented lesion region rather than collapsing toward the majority background. Boundary-aware loss enhances edge accuracy by computing spatial distances between predicted and ground truth boundaries, employing morphological gradient operator $\nabla = {\mathrm{Dilation}}(M) - {\mathrm{Erosion}}(M)$ to extract boundary pixel sets of segmentation masks, where dilation and erosion operations use *3 × 3* square kernels as structuring elements. The boundary loss is defined as

2 $${\mathcal{L}}_{{{\mathrm{Boundary}}}} = \frac{1}{|\partial G|}\sum\limits_{(x,y) \in \partial G} {\left\| {\nabla P(x,y) - \nabla G(x,y)} \right\|_{2}^{2} }$$

where *∂ G* represents the boundary pixel set of ground truth segmentation mask and *P* represents the prediction probability map. This loss function imposes additional spatial consistency constraints on boundary regions, promoting the network to learn precise edge localization capability, which is particularly critical for segmenting low-contrast lesions in ultrasound images.

The multi-scale deep supervision strategy adds auxiliary losses at intermediate decoder layers to supervise the network in learning hierarchical feature representations from coarse to fine. Outputs from the second, third, and fourth decoder stages are respectively mapped to auxiliary segmentation maps through *1 × 1* convolutions, with each auxiliary segmentation map computing Dice loss and binary cross-entropy loss against downsampled ground truth labels at corresponding resolutions. The study of medical image segmentation based on multi-axis attention proved the validity of multi-objective loss functions in improving image segmentation performance ^41^, which can address the problem of gradient vanishing in deep networks by adding supervision signals at different depths of the network. In this study, boundary-aware terms are proposed to improve the ability of edge localization for lesions with low contrast in ultrasound images. The total loss function is defined as

3 $${\mathcal{L}}_{{{\mathrm{Total}}}} = \lambda_{1} {\mathcal{L}}_{{{\mathrm{Dice}}}}^{{^{{{\mathrm{main}}}} }} + \lambda_{2} {\mathcal{L}}_{{{\mathrm{BCE}}}}^{{^{{{\mathrm{main}}}} }} + \lambda_{3} {\mathcal{L}}_{{{\mathrm{Boundary}}}} + \lambda_{4} \sum\limits_{j = 2}^{4} {({\mathcal{L}}_{{{\mathrm{Dice}}}}^{{^{(j)} }} + {\mathcal{L}}_{{{\mathrm{BCE}}}}^{{^{(j)} }} )}$$

where superscript main denotes the primary loss from the decoder’s final output and superscript *(j)* denotes the auxiliary loss from the *j*-th decoder stage. Weight coefficients are set as $\lambda_{1} = 0.5$, $\lambda_{2} = 0.3$, $\lambda_{3} = 0.1$, and $\lambda_{4} = 0.1$ experimentally, determined through grid search maximizing Dice coefficient on the validation set.

## Results

### Datasets and experimental configuration

The effectiveness of CEA-Net was validated on two public ultrasound image datasets. The BUSI dataset contains 780 breast ultrasound images covering three categories including benign tumors, malignant tumors, and normal tissue, with each image annotated by experienced radiologists providing lesion segmentation masks. The dataset was divided into training, validation, and test sets with a ratio of 7:1.5:1.5, resulting in 546 training images, 117 validation images, and 117 test images (Breast Ultrasound Images Dataset, BUSI, https://www.kaggle.com/datasets/aryashah2k/breast-ultrasound-images-dataset^42^. The TN3K dataset contains 3493 thyroid nodule ultrasound images provided by multiple medical institutions with strict quality control, divided into training, validation, and test sets with a ratio of 8:1:1, resulting in 2794 training images, 349 validation images, and 350 test images (Thyroid Nodule Segmentation Dataset, TN3K, https://github.com/haifangong/TRFE-Net-for-thyroid-nodule-segmentation^43^. The rationale for selecting these two datasets lies in their coverage of different anatomical organs with distinct imaging characteristics. Breast tissue in the BUSI dataset exhibits relatively high tissue contrast, while thyroid nodules in the TN3K dataset frequently present complex morphologies accompanied by calcification, cystic changes, and other complications. This diversity sufficiently validates the generalization capability and robustness of CEA-Net.

All images were normalized to the [0,1] interval and resized to a unified dimension of *256 × 256* pixels. Data augmentation strategies included random horizontal flipping (probability 0.5), random rotation ($\pm 15^\circ$), random scaling (0.9–1.1 times), and elastic deformation. These transformations were kept deliberately conservative so that each augmented image remains anatomically plausible, reproducing the variation in probe orientation, placement, and patient anatomy encountered in routine scanning without altering the diagnostic content or the correspondence with the segmentation mask. The narrow corner regions left empty by the rotation are filled with a constant value and are labelled as background in the correspondingly rotated mask, so that, covering only a small peripheral area, they introduce no spurious lesion structure and leave the segmentation of the central region unaffected. The experimental environment was configured with a cloud server equipped with an NVIDIA RTX 3090 GPU and an edge device based on the NVIDIA Jetson Nano Developer Kit, which integrates a 128-core Maxwell GPU and a quad-core ARM Cortex-A57 processor with 4 GB of LPDDR4 memory. The software framework consisted of PyTorch 1.12.1 and CUDA 11.3. Network training employed the AdamW optimizer with an initial learning rate of $1 \times 10^{ - 4}$, batch size of 16, and cosine annealing learning rate scheduling strategy that decayed the learning rate to $1 \times 10^{ - 6}$ over 200 epochs. Segmentation performance was evaluated through Dice coefficient, Intersection over Union (IoU), Precision, and Recall. The Dice coefficient is defined as

4 $${\mathrm{Dice}} = \frac{2|P \cap G|}{{|P| + |G|}}$$

and IoU is defined as

5 $${\mathrm{IoU}} = \frac{|P \cap G|}{{|P \cup G|}}$$

where *P* and *G* represent the predicted region and ground truth annotation region, respectively. Efficiency metrics include inference time, frames per second (FPS), number of parameters, and floating-point operations (FLOPs). Baseline methods include classical convolutional neural networks U-Net^44^, U-Net +  +^45^, and Attention U-Net^46^, Transformer-based methods TransUNet^47^, Swin-UNet^48^, and UNETR +  +^9^, as well as lightweight methods UNeXt^49^, MedNeXt^50^, USEANet^51^, and Wave-GMS^52^. All methods were trained with identical configurations to ensure fair comparison.

### Segmentation performance comparison

Comparative experiments on the BUSI dataset are conducted to verify whether the proposed CEA-Net can achieve or even surpass the accuracy of heavyweight models while remaining lightweight in the application of breast ultrasound image segmentation. The comparative experiments are conducted under strict control rules. The models are trained for 200 epochs on the same training set and selected on the validation set under identical configuration. The selection of the comparative models takes different technical routes from traditional convolutional networks to the latest Transformer networks to fully demonstrate the superiority of CEA-Net against different technical routes. The experiments are conducted to verify whether the proposed cloud-edge collaborative architecture can break through the accuracy bottleneck of traditional lightweight networks and whether the dynamic inference mechanism has a great influence on the quality of image segmentation, as indicated in Table 1. Given the limited size of the BUSI dataset, the results reported in Table 1 were obtained through five-fold cross-validation, in which the dataset was partitioned into five mutually exclusive folds with four used for training and the remaining one for testing in rotation, while the threshold θ and the loss weight coefficients were kept fixed at the values determined on the validation set of the primary split to prevent information leakage, so that every image was tested exactly once and the reported values denote the mean and standard deviation across the five test folds.

**Table 1:** Performance comparison with state-of-the-art methods on BUSI dataset.

Methods	Dice (%)	IoU (%)	Precision (%)	Recall (%)
U-Net	82.7 ± 0.6	70.6 ± 0.7	83.5 ± 0.6	82.0 ± 0.7
U-Net + +	84.1 ± 0.6	72.6 ± 0.6	84.9 ± 0.6	83.4 ± 0.6
Attention U-Net	85.3 ± 0.5	74.4 ± 0.6	86.1 ± 0.5	84.6 ± 0.6
TransUNet	86.2 ± 0.7	75.8 ± 0.8	89.1 ± 0.6	83.6 ± 0.9
Swin-UNet	87.1 ± 0.5	77.3 ± 0.5	88.0 ± 0.6	86.3 ± 0.6
UNeXt	84.6 ± 0.6	73.3 ± 0.7	85.3 ± 0.6	83.9 ± 0.7
UNETR + +	86.7 ± 0.6	76.6 ± 0.6	88.5 ± 0.5	85.2 ± 0.6
MedNeXt	87.4 ± 0.5	78.1 ± 0.5	88.1 ± 0.5	86.8 ± 0.5
USEANet	87.6 ± 0.5	78.3 ± 0.5	88.6 ± 0.6	86.9 ± 0.5
Wave-GMS	87.9 ± 0.4	78.7 ± 0.5	88.8 ± 0.5	87.2 ± 0.5
CEA-Net (Ours)	88.3 ± 0.5	79.2 ± 0.6	89.3 ± 0.5	87.8 ± 0.5

Values are reported as mean ± standard deviation over five-fold cross-validation.

As indicated in Table 1, the Dice coefficient value of the proposed CEA-Net model reaches 88.3%, which is 5.6% higher than U-Net and 1.2% higher than Swin-UNet. The IoU value reaches 79.2%, which is higher than the value of 78.1% achieved by the MedNeXt model. The lightweight encoder structure greatly eliminates the redundancy of model parameters, and the channel and space joint attention significantly improves the expression of boundary features. Although the accuracy rate of the TransUNet model reaches 89.1%, its recall rate only reaches 83.6%. The proposed model has a better balance in terms of accuracy rate and recall rate, with values of 89.3% and 87.8%, respectively, using a hybrid structure. CEA-Net also attains a higher Dice than the lightweight USEANet and Wave-GMS, reaching 88.3% against their 87.6% and 87.9%, which extends its accuracy advantage to these two networks as well. The narrow standard deviations obtained across the folds confirm that the margin separating CEA-Net from the competing networks on BUSI is consistent across partitions rather than contingent on a favourable split.

To verify the cross-domain generalization performance of the proposed CEA-Net model, the experiment used the TN3K dataset for validation tests. As the characteristics of thyroid nodules and breast tumors are different in terms of imaging features, morphological complexity, and boundary clearness, the experiment has higher demands on the robustness of the image segmentation algorithm. The experiment adopted the same training strategy, evaluation criteria, and five-fold cross-validation protocol used for the BUSI comparison, with the TN3K dataset partitioned into five mutually exclusive folds evaluated in rotation and the reported values denoting the mean and standard deviation across the five test folds, which collectively span thyroid nodules of varying size and shape.

As can be seen from Table 2, the value of the Dice coefficient of CEA-Net is 86.9%, and the value of the IoU is 77.0%, ranking the highest among the three indicators. The performance from BUSI to TN3K decreases by merely 1.4%, while TransUNet decreases by 2.8%. This further proves the stability of the hybrid structure across domains. Although the accuracy rate of TransUNet is high, reaching 87.8%, the recall rate is merely 79.8%. This is due to the missed detection of the Transformer structure in the complex morphology of the thyroid gland. The recall rate of the proposed CEA-Net is high, reaching 86.4%. This proves the superiority of the lightweight encoder and the dynamic reasoning structure in balancing the precision and recall of the network. The small standard deviations across the five folds show that this advantage on the limited TN3K dataset is stable rather than an artefact of a single partition. On the TN3K dataset CEA-Net likewise exceeds USEANet and Wave-GMS, reaching a Dice of 86.9% against their 86.2% and 86.4%, which confirms that this advantage also holds for thyroid nodule segmentation.

**Table 2:** Performance comparison with state-of-the-art methods on TN3K dataset.

Methods	Dice (%)	IoU (%)	Precision (%)	Recall (%)
U-Net	81.5 ± 0.5	68.8 ± 0.6	82.1 ± 0.7	80.9 ± 0.6
U-Net + +	82.8 ± 0.6	70.7 ± 0.5	83.5 ± 0.5	82.2 ± 0.7
Attention U-Net	83.7 ± 0.4	72.0 ± 0.6	84.8 ± 0.6	82.7 ± 0.5
TransUNet	83.4 ± 0.7	71.5 ± 0.8	87.8 ± 0.5	79.8 ± 0.9
Swin-UNet	85.3 ± 0.5	74.4 ± 0.4	86.2 ± 0.6	84.5 ± 0.5
UNeXt	83.2 ± 0.6	71.2 ± 0.7	84.1 ± 0.6	82.4 ± 0.7
UNETR + +	84.9 ± 0.6	73.8 ± 0.5	86.7 ± 0.5	83.4 ± 0.6
MedNeXt	86.1 ± 0.4	75.7 ± 0.5	86.9 ± 0.5	85.4 ± 0.4
USEANet	86.2 ± 0.5	75.9 ± 0.5	86.9 ± 0.6	85.7 ± 0.5
Wave-GMS	86.4 ± 0.4	76.2 ± 0.5	87.0 ± 0.5	85.9 ± 0.5
CEA-Net (Ours)	86.9 ± 0.4	77.0 ± 0.5	87.5 ± 0.6	86.4 ± 0.4

Values are reported as mean ± standard deviation over five-fold cross-validation.

In addition, it is necessary to comprehensively evaluate the complexity of the model and the efficiency of the inference to verify the superiority of the lightweight design of the proposed method. The inference performance of all the methods was tested on the cloud GPU platform and the CPU platform in the experiment. The batch size of all the methods was unified to be 1, simulating the frame-by-frame processing of the images in the real world. The performance of the edge device deployment was tested on the Jetson Nano platform, and the batch size of the images was set to be different values to verify the latency, memory, and power consumption of the network. The model was optimized by TensorRT to fully utilize the hardware acceleration ability of the device. The power consumption of the device was monitored in real-time by the external power meter, as shown in Table 3.

**Table 3:** Model efficiency and edge deployment performance.

Methods/configuration	Params (M)	FLOPs (G)	GPU Latency (ms)	CPU Latency (ms)	FPS	Memory (MB)	Power (W)
Model complexity comparison							
U-Net	31.0	54.3	18.7	142.5	53	–	–
U-Net + +	36.2	61.8	21.3	168.9	47	–	–
Attention U-Net	33.7	57.6	19.8	155.2	50	–	–
TransUNet	105.3	98.4	43.6	387.2	23	–	–
Swin-UNet	89.4	52.7	37.8	312.5	26	–	–
UNeXt	1.47	3.8	9.6	96.8	104	–	–
UNETR + +	89.5	67.3	41.2	358.7	24	–	–
MedNeXt	18.6	31.4	25.6	198.4	39	–	–
USEANet	3.64	0.79	14.2	118.3	70	–	–
Wave-GMS	2.60	4.7	13.5	108.6	74	–	–
CEA-Net (Ours)	0.83	2.1	11.8	89.4	85	–	–
Edge device deployment (Jetson Nano)							
CEA-Net (Batch = 1)	0.83	2.1	–	52.3	19	512	5.4
CEA-Net (Batch = 2)	0.83	2.1	–	98.7	20	697	6.2
CEA-Net (Batch = 4)	0.83	2.1	–	191.5	21	1138	7.1
CEA-Net (Batch = 8)	0.83	2.1	–	367.8	22	2015	8.3

GPU: NVIDIA RTX 3090; CPU: Intel i9-10900 K; Edge Device: NVIDIA Jetson Nano Developer Kit. FPS denotes frames (images) processed per second, computed as the batch size divided by the corresponding per-batch latency. “–” indicates not applicable.

Table 3 shows that the parameter quantity of CEA-Net, 0.83 M, is compressed by 97.3% compared to that of U-Net, 31.0 M. It is even lower than that of the lightweight method UNeXt, 1.47 M. The GPU latency for pure cloud-mode processing with complete 4-stage decoding is 11.8 ms, achieving 85 FPS. Although slightly slower than UNeXt’s 9.6 ms, CEA-Net achieves significantly higher segmentation accuracy (88.3% vs 84.6% Dice). The 0.83 M parameters of CEA-Net also remain below the 3.64 M of USEANet and the 2.60 M of Wave-GMS, and its GPU latency of 11.8 ms, shorter than the 14.2 ms of USEANet and the 13.5 ms of Wave-GMS, indicates a more compact and faster design than these two networks. When deployed in cloud-edge collaborative mode, CEA-Net achieves 8.3 ms weighted average latency (120 FPS) through intelligent task allocation, where 63.8% of simple samples are processed via 3-stage early exit at 6.5 ms and 36.2% of complex samples undergo full 4-stage processing at 11.8 ms, demonstrating superior efficiency-accuracy balance. The rise from 19 to 22 frames per second as the batch size grows from one to eight reflects the improved utilization of the parallel computing units on the Jetson Nano, whereas the lowest single-frame latency at a batch size of one makes that configuration preferable for real-time frame-by-frame scanning.

### Ablation study

The ablation study adopts an incremental approach for the addition of modules, starting from the basic U-Net structure, then incorporating the lightweight encoder, attention fusion module, and dynamic inference mechanism, in order to evaluate the contribution of each module by measuring the change in performance at each step of the ablation process. In incorporating the lightweight encoder, conventional convolution is replaced with depthwise separable convolution and inverted residual convolution, while the attention fusion module incorporates channel-spatial joint attention in each stage of the decoder network. The training process for all ablation models is the same, with the models trained on the BUSI dataset and evaluated under the five-fold protocol described above, with the number of parameters and latency measured for each model. The ablation studies on the TN3K dataset confirm the necessity of each component in the proposed model, as shown in Table 4, by removing a single module at a time. Following the same five-fold cross-validation protocol, the Dice and IoU values in Table 4 are reported as the mean and standard deviation across the five folds.

**Table 4:** Ablation study on the contribution of each module.

Configuration	Dice (%)	IoU (%)	Params (M)	Latency (ms)
Progressive addition on BUSI dataset				
Baseline (U-Net Structure)	79.2 ± 0.9	65.5 ± 1.0	31.0	18.7
+ Lightweight Encoder	83.5 ± 0.7	71.7 ± 0.8	0.75	9.8
+ Attention Module	85.8 ± 0.6	75.1 ± 0.7	0.83	11.3
+ Dynamic Inference	87.1 ± 0.6	77.2 ± 0.6	0.83	11.5
Full CEA-Net	88.3 ± 0.5	79.2 ± 0.6	0.83	11.8
Component removal on TN3K dataset				
w/o Dynamic Inference	85.1 ± 0.5	74.2 ± 0.6	0.83	11.6
w/o Attention Module	84.3 ± 0.6	72.8 ± 0.5	0.75	9.8
w/o Lightweight Encoder	81.8 ± 0.7	69.2 ± 0.8	31.0	18.3
Full CEA-Net	86.9 ± 0.4	77.0 ± 0.5	0.83	11.8

All latency measurements conducted on NVIDIA RTX 3090 GPU with fixed 4-stage decoding to ensure fair comparison across configurations. Dice and IoU are reported as mean ± standard deviation over five-fold cross-validation.

As can be seen from Table 4, lightweight encoder reduces 31.0 M parameters down to 0.75 M, while achieving 83.5% Dice and 9.8 ms GPU latency, up from 79.2% and 18.7 ms, thanks to the use of depthwise separable convolutions. The attention module contributes 2.3 percentage points with only 0.08 M additional parameters, and dynamic inference contributes 1.3 percentage points. The remaining 1.2 percentage points to achieve 88.3% is through the complete optimization of the multi-objective loss with $\lambda_{1} = 0.5$, $\lambda_{2} = 0.3$, $\lambda_{3} = 0.1$, $\lambda_{4} = 0.1$ and multi-scale deep supervision. The latency of all models is measured with fixed 4-stage decoding for fair comparison, while the operational cloud-edge collaborative mode reduces latency through adaptive routing. The degradation of 5.1 percentage points in TN3K by removing the lightweight encoder verifies its importance in achieving cross-dataset generalization. The small standard deviations across folds confirm that the incremental gain attributed to each module is consistent and not a by-product of a particular split.

### Cloud-edge collaborative inference validation

The verification experiment of the cloud-edge collaborative reasoning mechanism is intended to verify the superiority of the dynamic routing strategy compared to the fixed deployment mode, as well as the effectiveness of the sample complexity-aware algorithm. The experiment adopts a control scheme of pure cloud mode, pure edge mode, and cloud-edge collaborative mode. In the pure cloud mode, all the test samples are processed on the GPU server to ensure the highest accuracy, while in the pure edge mode, all the samples are processed on the Jetson Nano to ensure the lowest latency. In the cloud-edge collaborative mode, the processing path is dynamically chosen based on the comparison of the confidence score c and the threshold θ. The experiment measures the performance of the Dice coefficient, inference delay, and energy consumption of the three modes on the BUSI test set. The energy consumption is calculated through the device’s power consumption and processing time. The analysis of the statistics of the dynamic reasoning includes the proportion of simple samples and complex samples, the average performance of each type of sample, and the routing accuracy, as shown in Table 5. Consistent with the preceding experiments, the Dice scores and the routing accuracy in Table 5 are reported as the mean and standard deviation over five-fold cross-validation.

**Table 5:** Dynamic inference mechanism validation and performance under different cloud-edge modes.

Mode/sample type	Dice (%)	Latency (ms)	Proportion (%)	Routing accuracy (%)
Deployment mode comparison				
Cloud-only mode	88.3 ± 0.5	11.8	–	–
Edge-only mode	82.3 ± 0.8	6.5	–	–
Cloud-edge collaborative mode	87.1 ± 0.6	8.3	–	91.2 ± 1.0
Dynamic inference sample analysis				
Simple samples (edge processing)	88.9 ± 0.5	6.5	63.8	–
Complex samples (cloud processing)	84.2 ± 0.8	11.8	36.2	–
Weighted average performance	87.1 ± 0.6	8.3	100.0	–

All tests conducted on BUSI dataset. Cloud mode uses full 4-stage decoder, edge mode uses 3-stage early exit. Routing threshold θ = 0.7. Dice scores and routing accuracy are reported as mean ± standard deviation over five-fold cross-validation.

As shown in Table 5, the collaborative mode of cloud edge can reach a Dice of 87.1% with a weighted average latency of 8.3 ms. The latency of the collaborative mode is calculated based on the distribution of samples, where 63.8% of simple samples are processed in 6.5 ms through the 3-stage early exit strategy, and 36.2% of complex samples are processed in 11.8 ms through the complete 4-stage decoding strategy. Compared to the pure cloud mode, which can reach a Dice of 88.3% with a latency of 11.8 ms, the collaborative mode can reduce the latency by 29.7% while reducing the accuracy by merely 1.2%. The narrow standard deviations of the mode-wise Dice scores and of the routing accuracy across folds indicate that both the latency-accuracy trade-off and the reliability of the complexity-aware routing remain stable across data partitions.

### Visualization and qualitative analysis

**Fig. 5:**
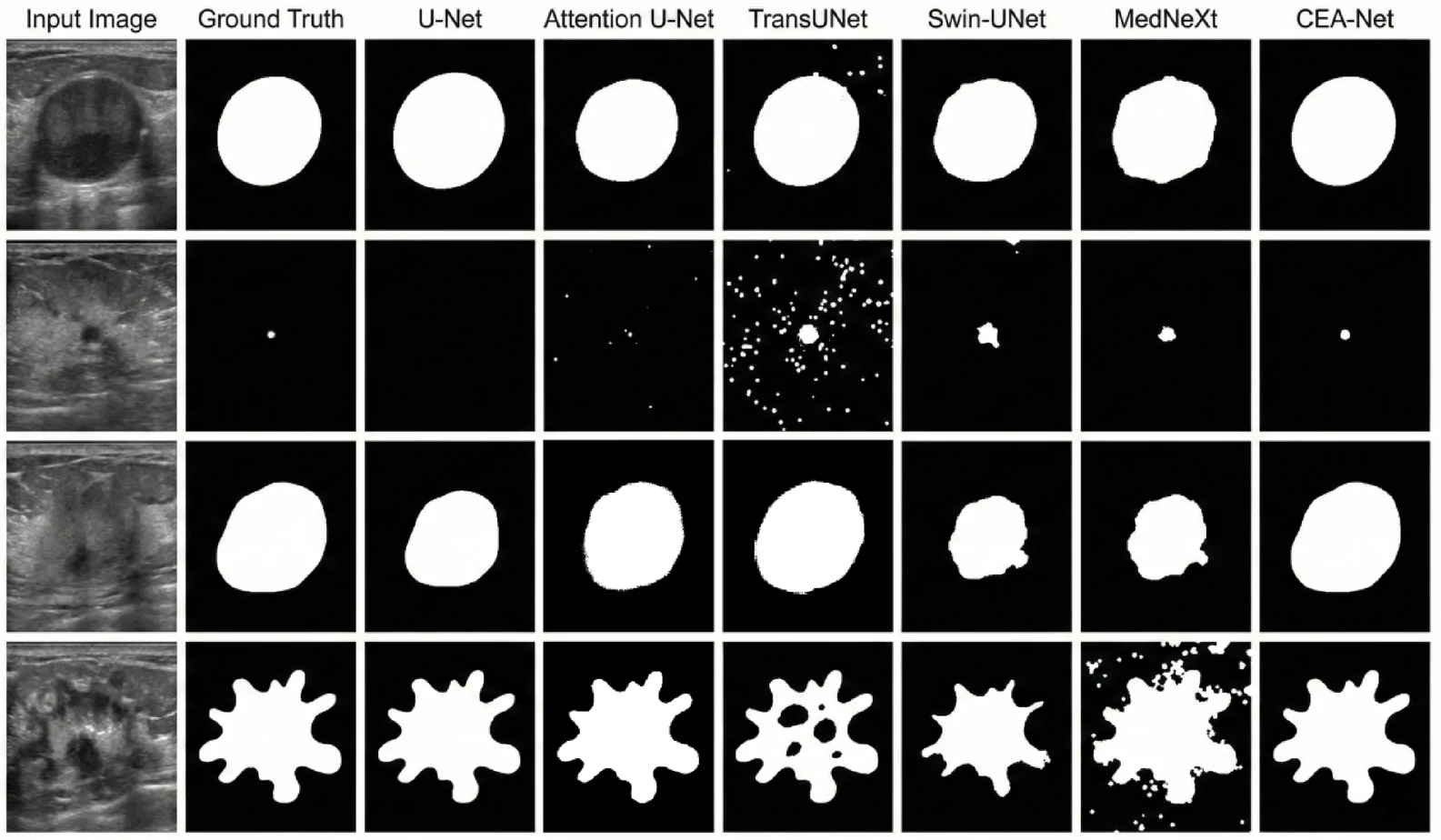
Qualitative segmentation results comparison on BUSI dataset.

Qualitative comparison on the BUSI dataset shows the performance of CEA-Net. Four cases of representative lesions are chosen from the test set: large regular lesions (Row 1), small deep lesions (Row 2), low contrast blurred boundaries (Row 3), and irregular heterogeneous textures (Row 4), as shown in Fig. 5. For fair visual comparison, the probability output of every method is binarized at a common threshold of 0.5 before rendering, so that the displayed differences reflect segmentation behaviour rather than display conventions.

Figure 5 shows the superiority of the proposed CEA-Net in accurately detecting the small target that U-Net fails to segment while TransUNet responds with widespread fragmented false positives (row 2), in recovering the low-contrast lesion that Swin-UNet and MedNeXt under-segment (row 3), and in preserving boundary integrity on the large regular lesion (row 1) without the internal holes (TransUNet, row 4) or the speckle over-segmentation (MedNeXt, row 4) seen in the other methods.

**Fig. 6:**
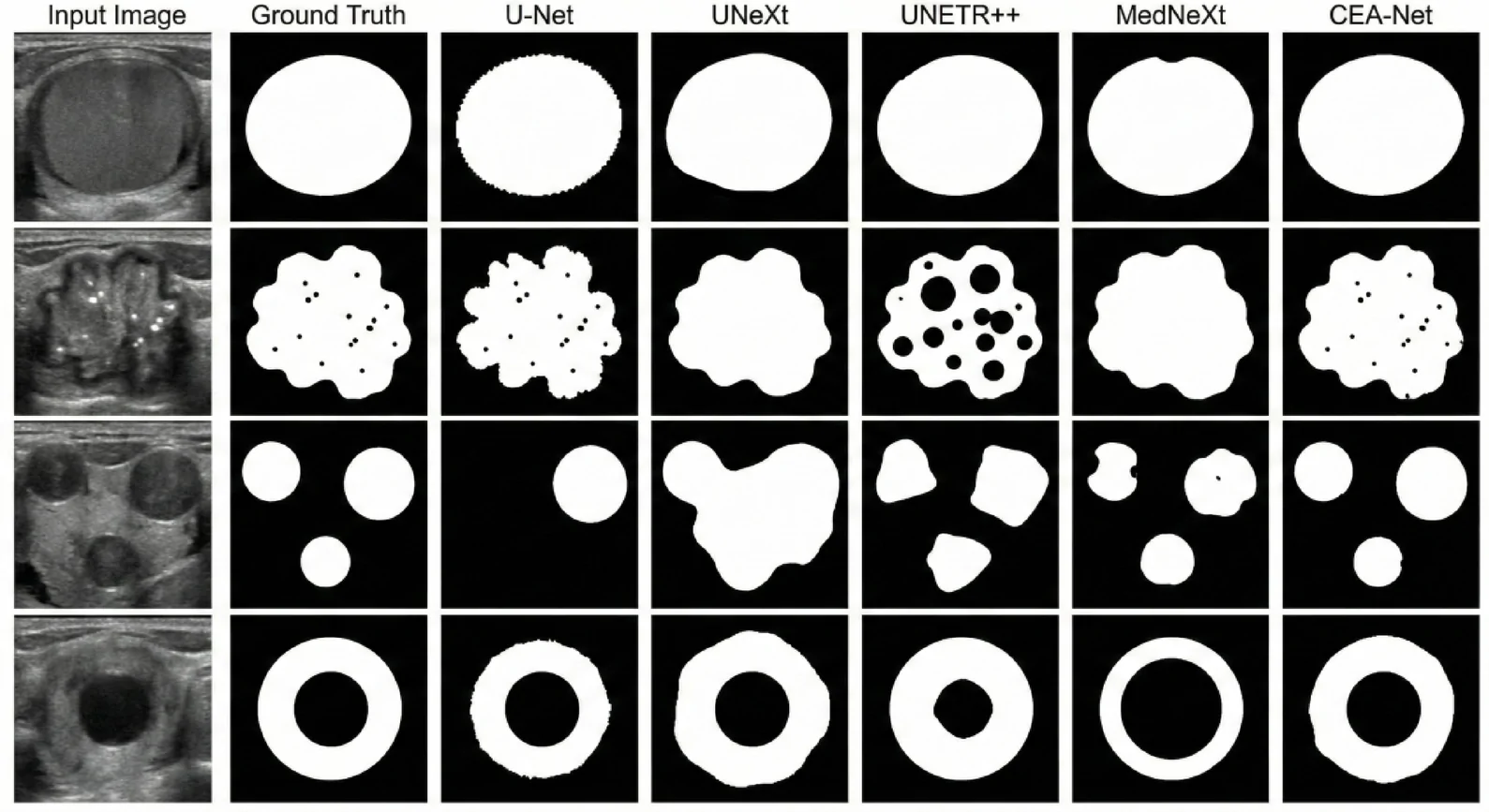
Qualitative segmentation results comparison on TN3K dataset.

Qualitative evaluation using the TN3K dataset assesses the performance of CEA-Net for complex thyroid nodule morphologies. Four typical cases include regular circular nodules (row 1), irregular calcified nodules (row 2), multiple small nodules (row 3), and ring-shaped cystic nodules (row 4) as shown in Fig. 6.

As shown in Fig. 6, for the first row of standard cases, all methods correctly detected the main body, but there were certain differences in the accuracy of boundaries. In the second row of calcification cases, UNETR +  + incorrectly recognized the large internal calcification as background, causing multiple large black holes to appear. In the third row of multiple nodules, U-Net recognized only one nodule, while the other methods incorrectly recognized multiple nodules or caused severe morphological distortion. In the fourth row of circular structures, the size of the central black hole detected by MedNeXt was significantly larger. In the four cases, CEA-Net had the best morphological accuracy and internal structure processing ability.

**Fig. 7:**
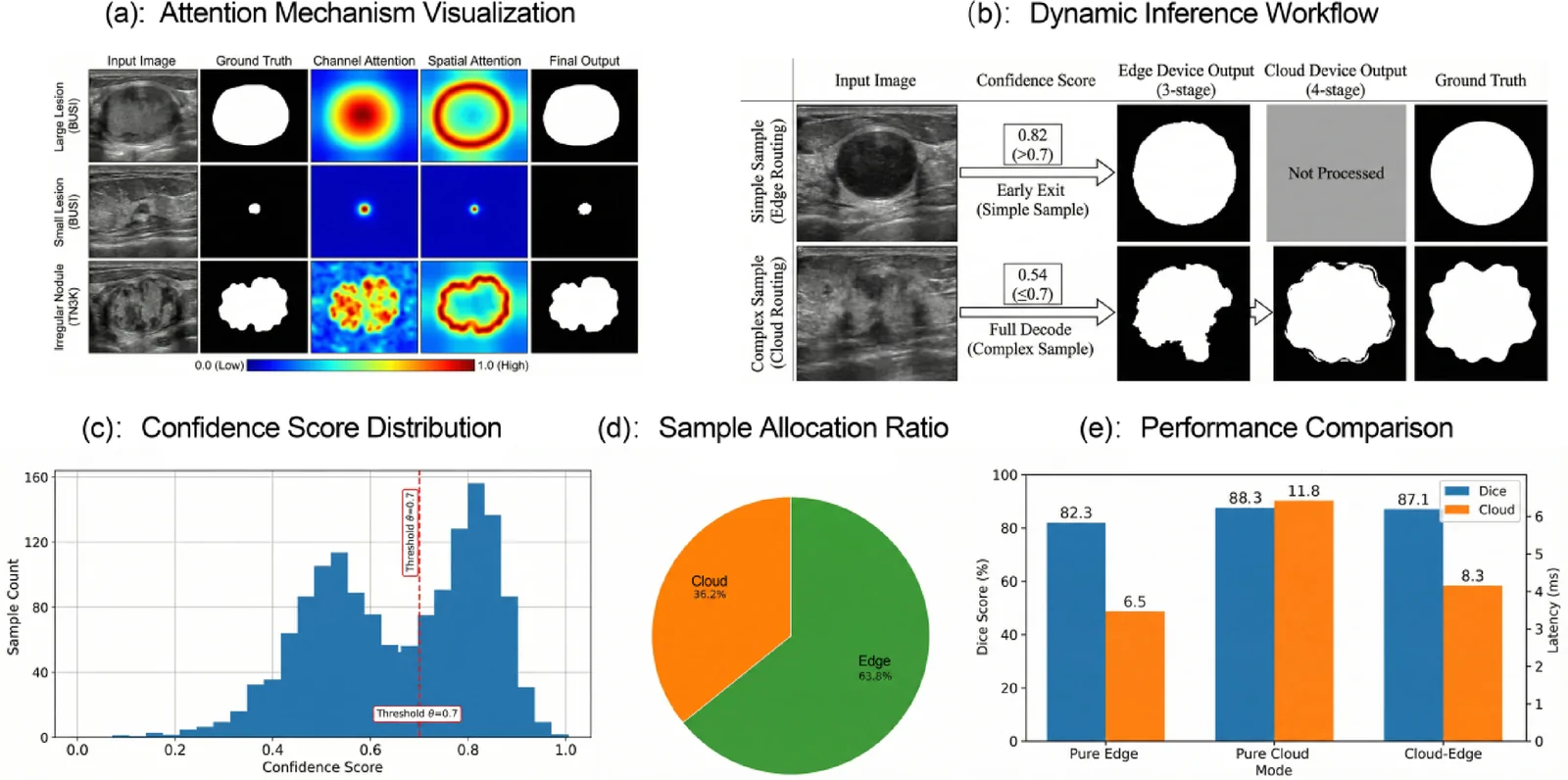
Attention mechanism visualization and cloud-edge dynamic inference workflow.

Visualization of the attention mechanisms and dynamic inference workflow can help to better understand the inner working mechanisms of CEA-Net. The attention maps can illustrate the channel-spatial attention focus on specific lesion areas. The dynamic inference can visualize the processing paths of simple and complex samples, including confidence calculation, routing, and final outputs. Statistical analysis can also be used to quantitatively display confidence distribution, allocation ratio of samples, and performance comparison of deployment modes, as shown in Fig. 7.

Figure 7a indicates that the high responses in the lesion area for feature enhancement are obtained through channel attention and spatial attention. Figure 7b indicates that simple samples undergo early exit in the edge, while complex samples require complete processing on the cloud. Figure 7c shows the bimodal curve, proving that the threshold of 0.7 separates the two types of samples well. Figure 7d indicates that 63.8% of the samples undergo processing on the edge. Figure 7e verifies the reduction in latency by 29.7% at the expense of 1.2 percentage points in accuracy for the cloud-edge collaboration mode. The collaboration verifies the effectiveness of the attention enhancement and dynamic routing.

## Discussion

This research has reached a Dice coefficient of 88.3% and 86.9% for BUSI and TN3K datasets, respectively, with only 0.83 M parameters. Knowledge distillation methods can be used for model compression, and the performance of these methods relies on large models’ performance in a pre-trained state^53^. This research avoided relying on external knowledge sources by directly optimizing depthwise separable convolution, which reduced parameters from 31.0 M to 0.75 M and improved Dice coefficient from 79.2% to 83.5%. Hybrid attention mechanisms can be used to improve feature representation capabilities in medical image segmentation tasks^54^. This research minimized parameter usage in attention modules by using shared MLP strategies and reached 19 FPS in edge devices. Lightweight multi-dimensional attention models proposed strategies for cross-scale feature interaction^55^, and this research built upon these strategies to propose dynamic inference mechanisms. The proposed cloud-edge collaborative mode reduced accuracy by only 1.2% compared to the pure cloud mode, while it reduced latency by 29.7%. Three-dimensional ultrasound image segmentation has powerful capabilities in medical image processing, and the parameters are usually in the range of tens of millions^56^. This research has shown only 1.4% degradation in performance on the TN3K dataset, while TransUNet has shown 2.8% degradation.

Adaptive optimization in deep neural network inference was used for efficient utilization of resources through adaptive adjustment of network depth^57^. In this study, early exit strategies were combined with cloud-edge collaborative architectures, with 63.8% of simple samples achieving an accuracy of 88.9% using early exit, and 36.2% of complex samples achieving an accuracy of 84.2% using complete processing, with routing accuracy reaching 91.2%. Cloud-edge collaborative architectures in heart failure ultrasound diagnosis have been used for achieving a balance in terms of response accuracy and response speed^58^. Sample complexity perception modules were used in this study, with an improvement in Dice coefficient by 4.8% using the cloud-edge collaborative mode compared to using the edge mode alone. Lightweight pathological diagnosis cloud-edge collaborative platforms have been used for distributed training in a privacy-preserving manner^59^. Computational efficiency was optimized in this study using dynamic mechanisms in the inference stage.

The communication requirement of a cloud-edge segmentation system is governed by the volume of data that each method transfers to the server, which is in turn determined by the size of the representation uploaded to the cloud. The baseline networks compared in this study contain no edge-side inference path, so that a cloud deployment of any of them transfers the complete 256 × 256 × 3 input image to the server for every case, which fixes the per-case uplink at the full size of that image for all of these methods. CEA-Net instead transfers only the encoder feature map of size 16 × 16 × C4, which, despite its larger channel dimension, holds about one third of the values contained in the input owing to the 16-fold spatial reduction along each axis, and it performs this transfer only for the complex cases routed to the cloud, which form the 36.2 percent reported above, while the 63.8 percent handled on the edge incur no transfer at all, so that the expected per-case uplink is only a fraction of the full-image transmission that the other methods require for every case. This reduction in the volume of data transmitted to the cloud eases the demand on the constrained uplink bandwidth typical of primary healthcare and mobile diagnostic networks.

Although the advantages of accuracy and efficiency were verified in the present research, there are some limitations in it. The sensitivity of dynamic inference systems to fluctuations in the quality of data inputs is an important consideration for the robustness of the system^60^, where the sample complexity perception module in the present research is based on the statistical characteristics of encoder features, with the accuracy of the confidence score likely to be reduced in the presence of severe noise interference in the input image, where the accuracy of the experimental routing is 91.2% but leaves room for the remaining 8.8% to be routed to suboptimal processing paths. Security mechanisms in edge intelligent systems have been proposed for the collaboration of edge intelligent systems with the cloud^61^, but in the present research the architecture does not fully address data privacy protection and does not yet model the dynamic fluctuation of uplink bandwidth or the adaptive feature compression required under varying network conditions.

## Conclusion

This study proposes the design of the CEA-Net, which is a Cloud-Edge Adaptive Lightweight Network with dynamic inference. The design uses a lightweight encoder with only 0.83 M parameters by using depthwise separable convolution and inverted residual structures. The design also uses serial channel-spatial attention to improve the representation ability of the features. Moreover, the design uses intelligent routing algorithms based on sample complexity perception to achieve the function of cloud-edge collaborative dynamic inference. The experiments using the BUSI dataset and the TN3K dataset validate the design’s ability to achieve a favourable balance among the three aspects of accuracy, speed, and parameters. The experiment also shows the deployment on the edge device to achieve near real-time inference and the reduction in inference latency with the collaboration between the cloud and the edge devices to achieve high segmentation accuracy.

This study proposes a feasible solution for the practical deployment of the ultrasound image segmentation technology. The design’s lightweight architecture reduces the reliance on high-performance computing platforms, and the design’s dynamic inference mechanisms optimize the utilization efficiency of the computational resources. The design’s technical support for the application scenarios includes the practical deployment in the fields of real-time ultrasound diagnosis in primary healthcare institutions, remote medical consultation services, mobile medical vehicles, and so on. The design’s potential for future research includes the enhancement of the robustness of the algorithms for sample complexity perception for extremely low-quality images, the exploration of multi-modal ultrasound data fusion to expand the application scope, and the optimization of the cloud-edge communication to reduce the reliance on the network conditions.

## Data Availability

The datasets used in this study are publicly available. The BUSI (Breast Ultrasound Images Dataset) is available at https://www.kaggle.com/datasets/aryashah2k/breast-ultrasound-images-dataset. The TN3K (Thyroid Nodule Segmentation Dataset) is available at https://github.com/haifangong/TRFE-Net-for-thyroid-nodule-segmentation.
